# Morphometry Results of Formed Osteodefects When Using Nanocrystalline CeO_2_ in the Early Stages of Regeneration

**DOI:** 10.1155/2019/9416381

**Published:** 2019-12-26

**Authors:** Anton V. Lukin, Galina I. Lukina, Alexey V. Volkov, Alexander E. Baranchikov, Vladimir K. Ivanov, Alexey A. Prokopov

**Affiliations:** ^1^A. I. Evdokimov Moscow State University of Medicine and Dentistry, Moscow, Russia; ^2^N. N. Priorov National Medical Research Center of Traumatology and Orthopedics, Moscow, Russia; ^3^Kurnakov Institute of General and Inorganic Chemistry of the Russian Academy of Sciences, Moscow, Russia

## Abstract

This paper studies of the use of nanocrystalline cerium dioxide with artificially formed bone tissue defects. The results of morphometry confirmed the antialterative effect in the early stages of the reparative process of damaged bone tissue. When using calcium hydroxide with nanodispersed cerium dioxide, the nature of osteogenesis should be characterized as activated. In case of damage to the dentin of the roots of the teeth, dentinogenesis in presence of CeO_2_ occurs with the formation of a combined dentin and bone regenerates. Little or no studies of dentinogenesis in presence of CeO_2_ were performed by other researchers.

## 1. Introduction

Stimulation of bone tissue regeneration is an urgent problem of modern medicine. Solution strived for is the creation of optimal conditions for reparative osteogenesis and the prevention of pathological bone tissue regeneration. Optimization of reparative bone tissue regeneration processes is of key importance, and the search for new materials that contribute to the creation of optimal conditions for the formation of bone tissue remains relevant [1–4].

Recent researches and studies have found unique biochemical properties of nanocrystalline cerium dioxide. In vitro experimental studies have shown that cerium dioxide, CeO_2_, provides improved cell proliferation and provides antibacterial and antiviral effect [3–7, 8].

CeO_2_ is a unique inorganic material that exhibits a high degree of oxygen nonstoichiometry in the nanocrystalline state due to the presence of vacancies in the oxygen sublattice [2, 3, 6].

CeO_2_ has an advantage over existing antioxidants and, in some cases, surpasses them in its activity.

The catalase-like activity of nanocrystalline cerium dioxide depends on the content of cerium (III) ions on the surface of CeO_2_ particles and on the pH of the medium (with an increase in pH >7, the catalase-like activity increases and decreases with decreasing pH). In acidic media at pH <6, nanocrystalline CeO_2_ exhibits the properties of oxidoreductases [3, 4].

The ability of nanocrystalline CeO_2_ to perform the functions of enzymes represents an undeniable perspective when arresting all sorts of pathological processes associated with oxidative stress. Thus, cerium dioxide nanoparticles accelerate wound healing by reducing oxidative damage to membranes and proteins and increase the proliferation and migration of fibroblasts, keratinocytes, and vascular endothelial cells [2, 3, 6].

There is evidence that CeO_2_ nanoparticles affect the function of proliferation, differentiation, and mineralization of primary osteoblasts in vitro. The rate of these processes increases with decreasing of the size of CeO_2_ nanoparticles. It was shown that CeO_2_ nanoparticles do not have an acute cytotoxic effect on osteoblasts and can contribute to osteogenic differentiation and mineralization of osteoblasts [3, 6].

The purpose of this study is to determine, by morphometric parameters, the properties of nanocrystalline CeO_2_ in formed bone defects in the early stages of regeneration.

## 2. Materials and Methods

The study was conducted on 21 rabbits of the chinchilla breed. The animals were kept in accordance with the requirements of GOST R of 02.12.2009 534343-2009 “Principles of Good Laboratory Practice” (GLP). A soft tissue incision was made along the lower edge of the body of the mandible, up to 2 cm long. The incision was made under general anesthesia with the “Zoletil” drug and approved for official use in veterinary medicine, at the rate of 10 mg/kg body weight (intramuscular). The wool was sheared, and the operative field was treated with 5% iodine solution.

A hole with a diameter of 4-5 mm and a depth of about 4 mm was trepanned with use of saline solution chilled carbide bur. After treatment with 3% hydrogen peroxide and drying, an aqueous (isotonic solution) suspension containing calcium hydroxide (41%), barium sulfate (8%), and nanocrystalline cerium dioxide (80 mg in 5 ml) was placed in the mandible-shaped perforation hole of the lower jaw on one side (experimental side) to accelerate bone regeneration (in a volume corresponding to the volume of the defect (0.03 ml)). Wounds were sutured. On the other side (control side) of the lower jaw, access to the body of the lower jaw was formed in the same way, a perforation hole was formed, and a suspension of calcium hydroxide (0.03 ml volume) was placed. Wounds were sutured.

Animals were taken from the experiment in accordance with the provisions of the European Convention for the Protection of the Rights of Vertebrate Animals. Animals were removed using the drug “Zoletil” and approved for official use in veterinary medicine, at the rate of 10 mg/kg body weight (intramuscularly) after a certain period of time (2 weeks and 4 weeks) (according to the protocol). Furthermore, fragments of the operated bone tissue were extracted, and morphological (histological) studies were performed.

### 2.1. Histological Study

The bone tissue fragments were demineralized in 4% formic and hydrochloric acid solution within 48 hours, after which standard paraffin embedding was performed. Slices 5 *μ*m thick were made of bone tissue blocks, which were subsequently stained with hematoxylin and eosin, as well as Mallory stain. Histological samples were placed in a Leica mounting medium.

The study of histological samples and their photo documentation was carried out using a Leica 1500 DM microscope and an EC5 digital camera.

### 2.2. Morphometric Study

Morphometric study was performed according to the following principles:

For a period of 2 weeks,

  nBV—relative bone volume: newly formed bone tissue and lamellar bone tissue of interalveolar septa (tr.BV)
  MatV—the relative volume of the material
  IIV—the relative volume of granulation tissue
  Th.V—the relative volume of periodontal structures, including the root of the tooth
  Th.Tr—the average thickness of the bony part between the alveolar septum

For a period of 4 weeks,

  BV—the relative volume of bone tissue (the newly formed bone tissue was not excreted, since by this observation period, it was not possible to separate the maternal bone tissue and the newly formed bone tissue)
  MatV—the relative volume of the material
  IIV—the relative volume of granulation tissue
  Th.V—the relative volume of periodontal structures, including the root of the tooth
  Th.Tr—the average thickness of the bony part between the alveolar septum
  a—calcium hydroxide suspension
  k—calcium hydroxide suspension with nanodispersed cerium dioxide

Morphometric study was carried out in the software package MegaMorph12 (Russia).

### 2.3. Statistical Data Processing Methods

Processing of the results was carried out in the SigmaStat program. After testing of the obtained data for the normal distribution, one-sided dispersion analysis of Kruskal–Wallis was used and the Mann–Whitney method was used for pairwise intergroup differences. To determine the reliability of intergroup differences, a value of *p* equal to or less than 0.05 was taken.

## 3. Results

### 3.1. Histological Examination

**Figure 1:**
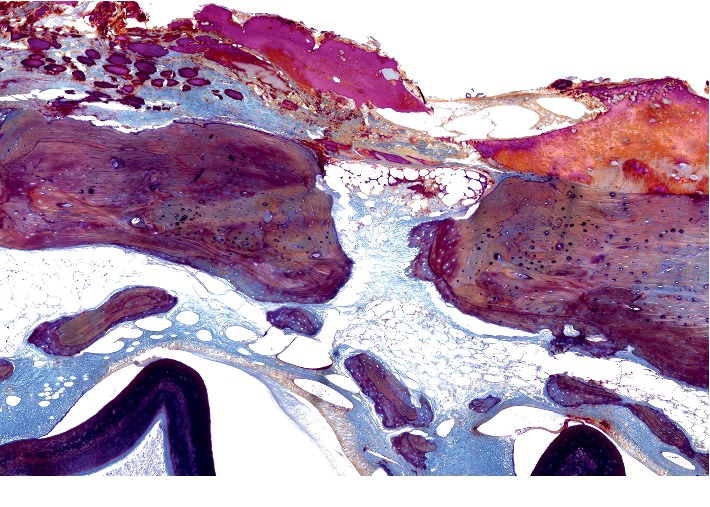
Histotopogram of the rabbit's mandible cross section, 2 weeks after the formation of a bone defect and placement of calcium hydroxide suspension.

**Figure 2:**
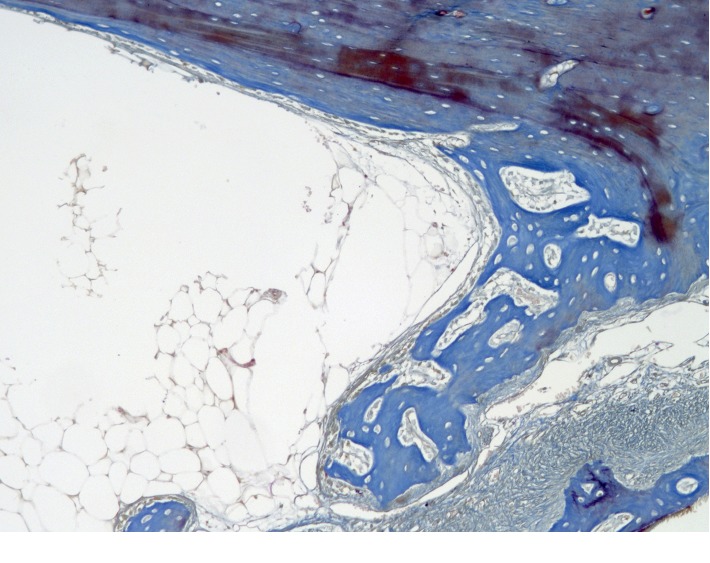
Cross section of the rabbit's jaw 2 weeks after the formation of a bone defect and introduction of calcium hydroxide suspension.

**Figure 3:**
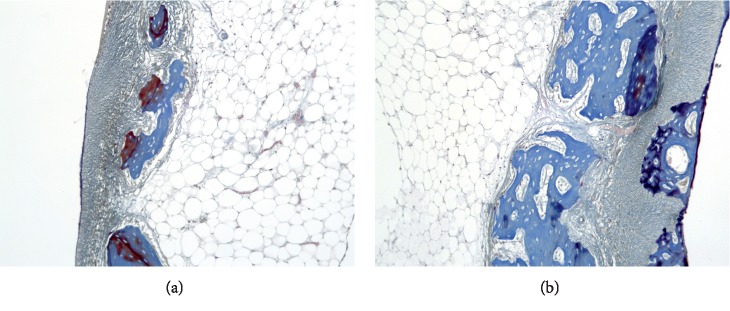
(a, b). Cross section of a rabbit's jaw 2 weeks after the formation of a bone defect and introduction of calcium hydroxide suspension.

**Figure 4:**
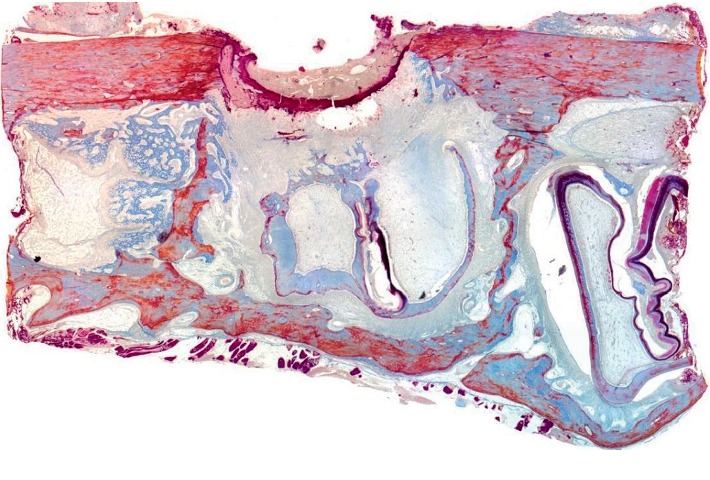
Histotopogram of the rabbit's mandible cross section 2 weeks after the formation of a bone defect and introduction of a suspension of calcium hydroxide with nanodispersed cerium dioxide.

*The state of bone tissue after 2 weeks after the formation of a bone defect and introduction of a suspension of calcium hydroxide* (Figures 1–4).

Figure 1 shows a fragment of the rabbit's mandible with an outer and an inner cortical plate, with a yellow bone marrow and the roots of the teeth in between. The outer cortical plate has a bone defect penetrating through it. The central part of the defect is filled with loose, fibrous connective tissue of the regenerative type and an amorphous substance. Deposits of amorphous masses with infiltration by macrophages are traced in the soft tissues over the defect. Macrophages are also found among the connective tissue of the regenerative type.

Under the periosteum on the surface of the cortical plate, newly formed bone tissue in the form of irregular outgrowths is detected. On the part of the endosteum, reticulofibrous bone formation is detected.

Alveolar plates are represented by a compact bone. In the area of the defect on the plates are found layers of osteoid and in the immediate vicinity—the formation of reticulofibrous bone tissue.

On the part of the endosteum (Figure 2), foci of reticulofibrous bone tissue are found. A large number of osteoblasts were detected on the surface of the developing bone structures. The newly formed bone structures are surrounded by an immature connective tissue of the regenerative type. The tissue is characterized by fine fibrous connective tissue matrix with spindle-shaped fibroblast-like cells and immature vessels.

The bone structures of the alveolar septa of the teeth are represented by lamellar bone tissue (Figure 3). Alveolar septa have through-holes and breaks, in which vessels and nerve trunks are found.

On some parts of the surface of the septa, facing the bone marrow, the endosteum has signs of the formation of reticulofibrous tissue on its surface in the form of irregular outgrowths. Some fragments of the lamellar bone have the phenomena of restructuring. No signs of osteogenesis have been identified in the periodontal area. Damage to the dentin of the roots of the teeth does not lead to pronounced stimulation of dentinogenesis. The periodontal tissue is thickened in places, with areas of formation of connective tissue of the regenerative type.

**Figure 5:**
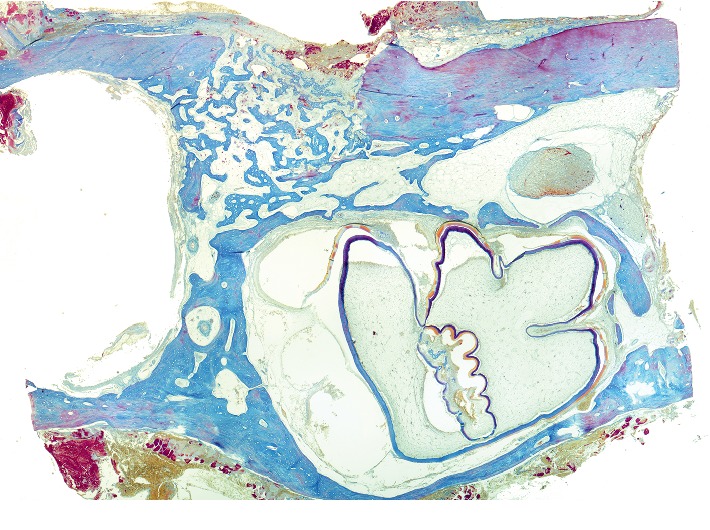
Histotopogram of the rabbit's mandible cross section 2 weeks after the formation of a bone defect and introduction of a suspension of calcium hydroxide with nanodispersed cerium dioxide.

**Figure 6:**
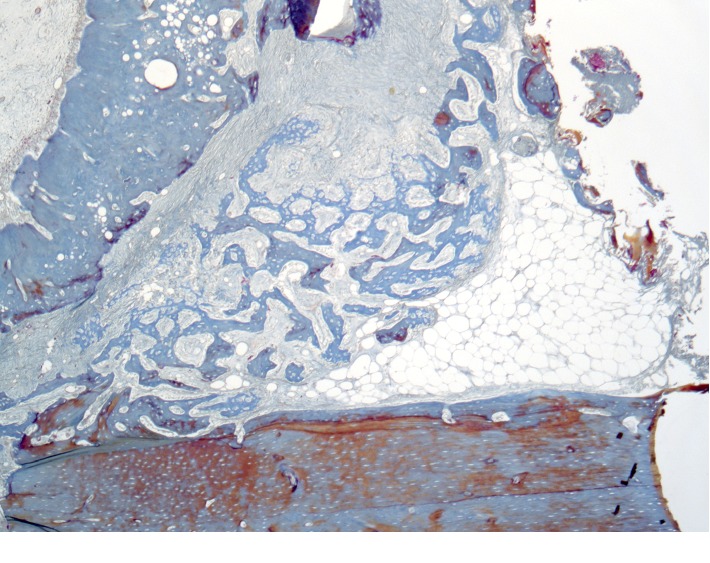
Histotopogram of the rabbit's mandible cross section 2 weeks after the formation of a bone defect and introduction of calcium hydroxide suspension with nanodispersed cerium dioxide.

**Figure 7:**
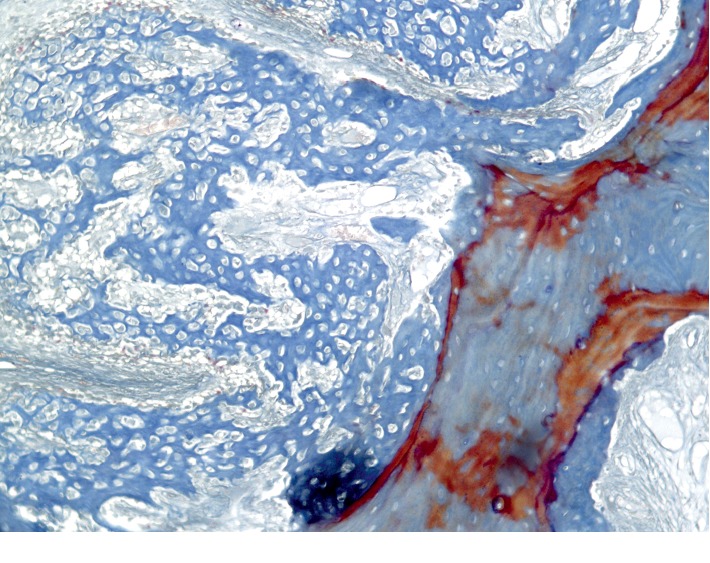
Histotopogram of the rabbit's mandible cross section 2 weeks after the formation of a bone defect and introduction of a suspension of calcium hydroxide with nanodispersed cerium dioxide.

**Figure 8:**
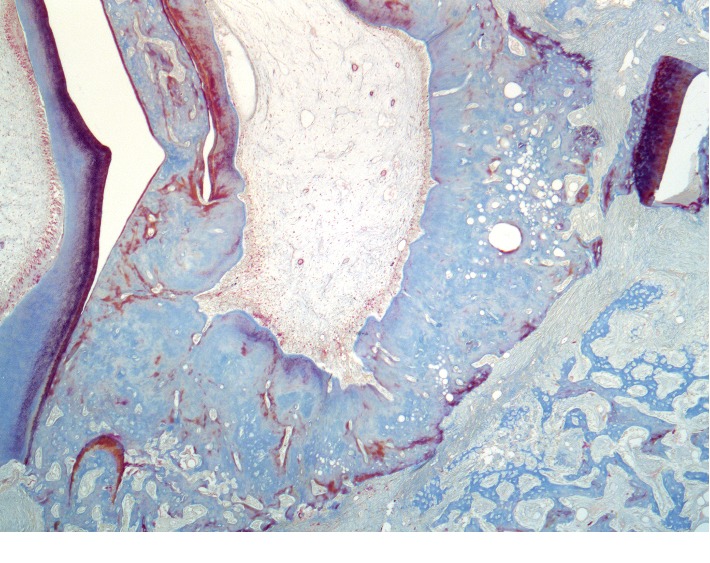
Histotopogram of the rabbit's mandible cross section 2 weeks after the formation of a bone defect and introduction of a suspension of calcium hydroxide with nanodispersed cerium dioxide.

The state of bone tissue 2 weeks after the formation of a bone defect and introduction of a suspension of calcium hydroxide with nanodispersed cerium dioxide (Figures 4–8).

The micrograph (Figure 4) shows a fragment of the lower jaw of a rabbit with an outer and an inner cortical plate, between which there is a yellow bone marrow and the roots of the teeth. The outer cortical plate has a bone defect penetrating through it. The central part of the defect is filled with loose, fibrous connective tissue of the regenerative type and an amorphous substance. Deposits of amorphous masses with macrophage infiltration are visible in the soft tissues above the defect. Macrophages are also found among the connective tissue of the regenerative type.

The newly formed bone tissue in the form of irregular projections is subperiosteal (Figure 5) on the surface of the cortical plate. On the side of the endosteum, reticulofibrous bone tissue is found to be formed with a tendency to close the bone defect.

Alveolar septa are represented (Figure 6) by a compact bone with a pronounced stratification of newly formed bone tissue. In the area of the defect, osteoid strata are found. In the immediate vicinity of the osteoid, the formation of reticulofibrous bone tissue and osteogenesis foci of the osteoid type are found.

In the periodontal region (Figure 7), signs of active osteogenesis are observed. The newly formed bone tissue has a reticulofibrous structure with foci of bone matter compaction. As a result of this, the bony plates of the interalveolar septa are sharply thickened.

Damaged dentin of the roots of the teeth shows signs of bone replacement in combination with the formation of foci of dentinogenesis (Figure 8). Dentinogenesis is being completed by the formation of an array of primary dentin without a clear transition into reticular fibrous bone tissue.

**Figure 9:**
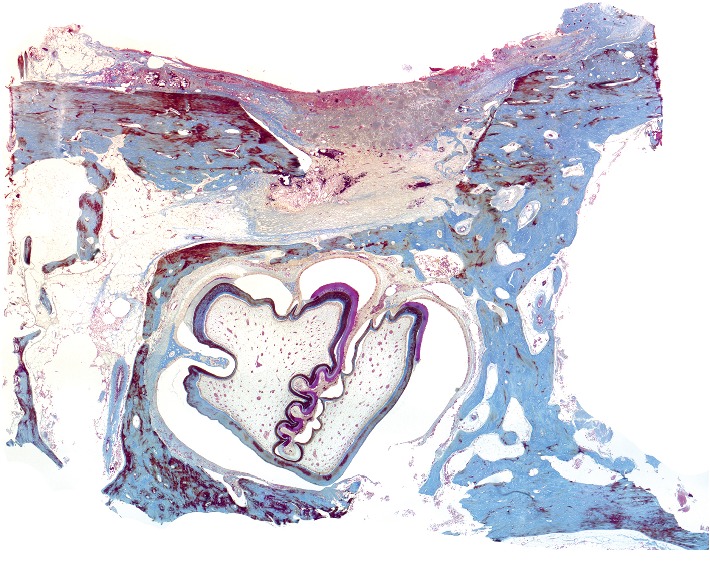
Histotopogram of a cross section of the mandible of the rabbit 4 weeks after the formation of a bone defect and introduction of calcium hydroxide suspension.

**Figure 10:**
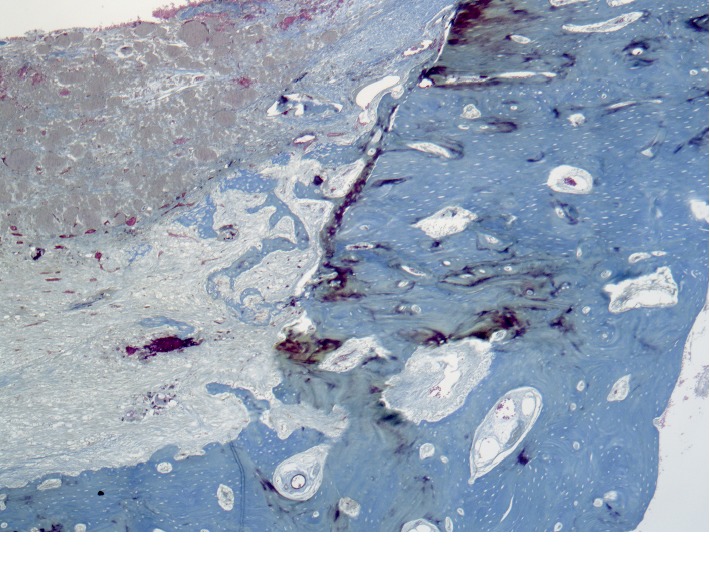
Histological picture of the cross section of the mandible of the rabbit 4 weeks after the formation of a bone defect and introduction of calcium hydroxide suspension.

**Figure 11:**
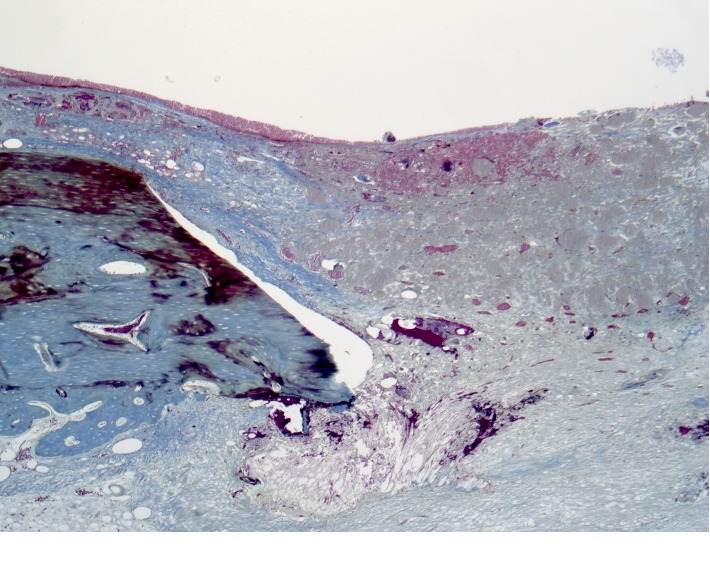
Histological picture of the cross section of the mandible of the rabbit 4 weeks after the formation of a bone defect and introduction of calcium hydroxide suspension.

**Figure 12:**
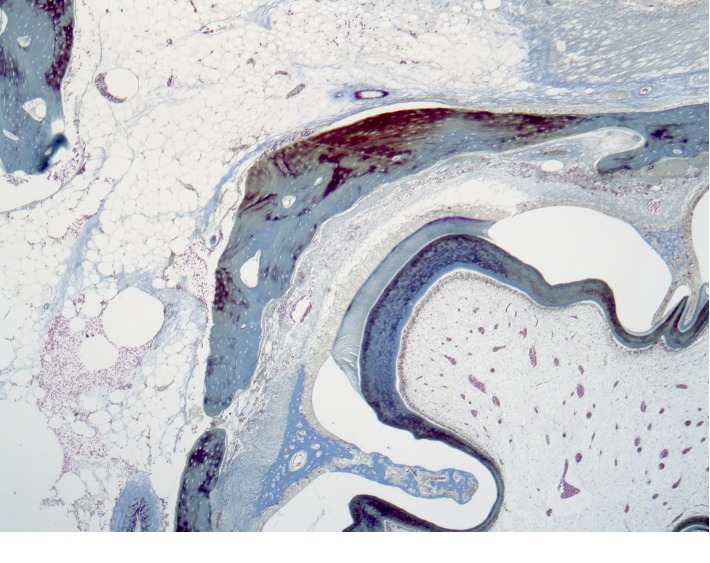
Histological picture of the cross section of the mandible of the rabbit 4 weeks after the formation of a bone defect and introduction of calcium hydroxide suspension.

The state of bone tissue 4 weeks after the formation of a bone defect and introduction of a suspension of calcium hydroxide (Figures 9–12).

Figure 9 shows a fragment of the rabbit's lower jaw with an outer and an inner cortical plate, between which there is a yellow bone marrow and the teeth roots. The outer cortical plate is with a bone defect penetrating through it. The central part of the defect is filled with loose, fibrous connective tissue of the regenerative type with amorphous substance, as well as with dense fibrous connective tissue. Deposits of amorphous masses with infiltration by macrophages were found in soft tissues over the defect. Macrophages are also found among the connective tissue of the regenerative type.

The newly formed bone tissue is present under periosteal, on the surface of the cortical plate. This new bone matter is in the form of irregular outgrowths (Figures 10–12). On the side of the endosteum, the bone trabeculae are represented by mineralized lamellar bone tissue. The small proportion of reticulofibrosis tissue was detected in the composition of the bone regenerate. Alveolar plates are represented by a compact bone. In the area of the defect, they are somewhat thickened but have a large number of fissures and through-holes. In the immediate vicinity of the defect, a similar histological pattern is observed. In the area of periodontal, there are signs of single foci of reparative osteogenesis.

**Figure 13:**
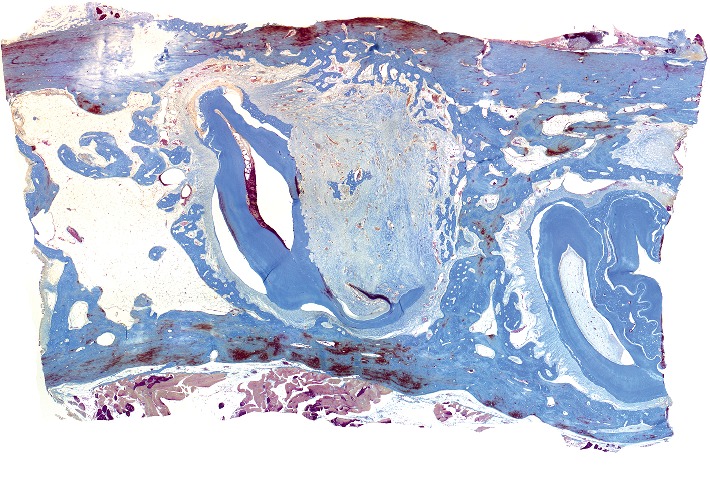
Histotopogram of the rabbit's mandible slice 4 weeks after the formation of a bone defect and introduction of a suspension of calcium hydroxide with nanodispersed cerium dioxide.

**Figure 14:**
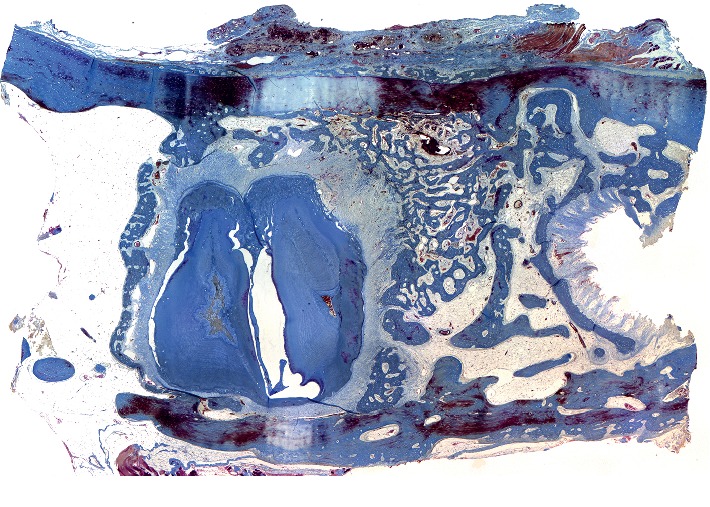
Histotopogram of the rabbit's mandible cross section 4 weeks after the formation of a bone defect and introduction of calcium hydroxide suspension with nanodispersed cerium dioxide.

**Figure 15:**
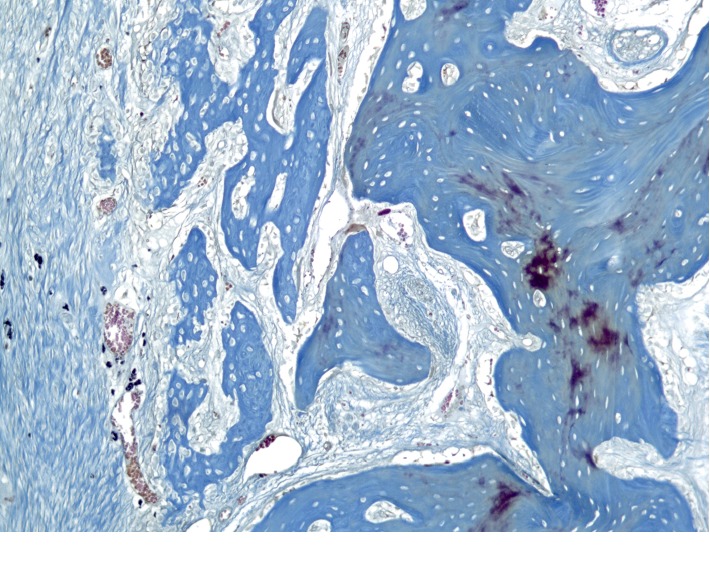
Histotopogram of the rabbit's mandible cross section 4 weeks after the formation of a bone defect and introduction of a suspension of calcium hydroxide with nanodispersed cerium dioxide.

The state of the bone tissue 4 weeks after the formation of a bone defect and introduction of a suspension of calcium hydroxide with nanodispersed cerium dioxide (Figures 13–15).

A microphotograph (Figure 13) presents a fragment of the rabbit's lower jaw with an outer and an inner cortical plate, between which there is a yellow bone marrow and the roots of the teeth. The outer cortical plate has a bone defect penetrating through it. The central part of the defect is filled with loose, fibrous connective tissue of the regenerative type and an amorphous substance. Deposits of amorphous masses with infiltration by macrophages were revealed in the soft tissues above the defect. Macrophages are also found among the connective tissue of the regenerative type.

Under periosteal, on the surface of the cortical plate, is a newly formed bone tissue in the form of irregular outgrowths (Figure 14). The defect area is filled with mineralized lamellar bone tissue. On the side of the endosteum, the formation of reticulofibrous bone tissue continues with a tendency to full-layer closure of the bone defect. The alveolar plates are sharply thickened and are represented by a compact bone with a pronounced accumulation of newly formed bone tissue and osteoid fields.

In the area of periodontal signs of active osteogenesis (Figure 15). The newly formed bone tissue has a reticulofibrous structure with foci of bone matter compaction. As a result, the bony plates between the alveolar septa are sharply thickened.

**Figure 16:**
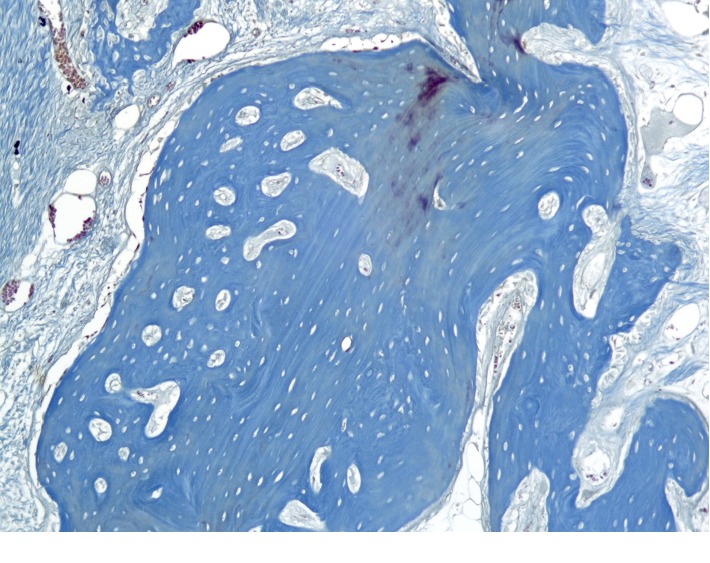
Histotopogram of the rabbit's mandible cross section 4 weeks after the formation of a bone defect and introduction of a suspension of calcium hydroxide with nanodispersed cerium dioxide.

Damaged dentin of the roots of the teeth shows signs of bone replacement in combination with the formation of foci of dentinogenesis (Figure 16). Dentinogenesis is completed by the formation of an array of primary dentin without a clear transition into reticular fibrous bone tissue.

### 3.2. The Results of the Morphometric Study

The structure of the cortical plate regenerated bone tissue 2 weeks after the formation of a bone defect and introduction of a suspension of calcium hydroxide and calcium hydroxide with nanodispersed cerium dioxide.

**Figure 17:**
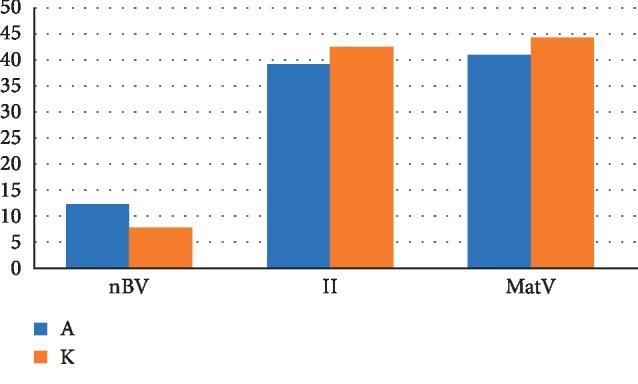
The distribution chart of the structural components of the bone regenerate of the cortical plate 2 weeks after the formation of a bone defect and introduction of calcium hydroxide suspension (both, with and without nanodispersed cerium dioxide). Where nBv is the relative volume of the newly formed bone tissue (%), II is the relative volume of the granulation tissue (%), and MatV is the relative volume of the material (%) (A with cerium dioxide and K with no cerium dioxide).

The chart (Figure 17) shows the difference in the distribution of the structural components of the cortical plate regenerated bone tissue 2 weeks after the formation of a bone defect and introduction of a suspension of calcium hydroxide alone and calcium hydroxide with nanocerium dioxide. The relative volume of the newly formed bone tissue (nBV%)in the presence of nanodispersed cerium dioxide 1.6 times (with the difference of 4.5%) exceeds the one with no nanodispersed cerium dioxide (nBV_a_ = 7.8%, nBV_k_ = 12.3%). The relative volume of granulation tissue (II %) formed in the bone defect in the presence of nanodispersed cerium dioxide is 1.08 (with the difference of 3.3%) lower than in defects without cerium dioxide (with a difference of 3.3%) (II_a_ = 39.2%, II_k_ = 42.5%). Relative volume of the injected into the defect material with cerium dioxide (MatV%) is 1.08 (with the difference of 3.3%) less than the material without cerium dioxide (MatV_a_ = 41%, MatV_k_ = 44.3%), which shows more active material absorption processes in the presence of nanocrystalline cerium dioxide in the early stages of regeneration.

The structure of regenerated cancellous bone tissue 2 weeks after the formation of a bone defect and introduction of suspensions of calcium hydroxide alone and calcium hydroxide with nanodispersed cerium dioxide.

**Figure 18:**
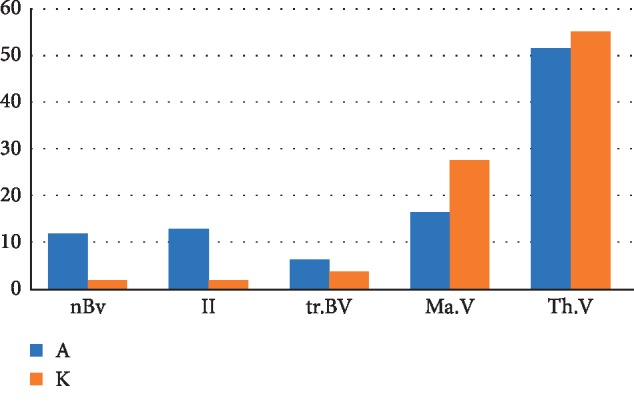
The distribution chart of the structural components of regenerated cancellous bone tissue 2 weeks after the formation of a bone defect and introduction of calcium hydroxide suspension (both, with and without nanodispersed cerium dioxide). Where nBv is the relative volume of the newly formed bone tissue (%), tr.BV is the relative volume of the lamellar bone tissue (old) (%), II is the relative volume of the granulation tissue (%), Ma.V is the relative volume of the bone marrow (%), and Th.V is the relative volume of the tooth and periodontal tissues (%) (A—with cerium dioxide and K—without cerium dioxide).

The chart (Figure 18) shows the difference in the distribution of the structural components of the regenerated cancellous bone tissue 2 weeks after the formation of a bone defect and introduction of a suspension of calcium hydroxide alone and calcium hydroxide with nanocerium dioxide. The relative volume of newly formed bone tissue in the presence of nanodispersed cerium dioxide significantly (6.6 times) exceeds that without it, which is 10.1% higher in volume (nBV_a_ = 11.9%, nBV_k_ = 1.8%). The relative volume of granulation tissue formed in the bone defect in the presence of nanodispersed cerium dioxide is 7.1 times higher than in defects without it by 11% (II_a_ = 12.8%, II_k_ = 1.8%). The relative volume of the lamellar bone tissue (old) is 1.8 times higher than that without it, which is 2.6% higher than relative volume (tr.BV_a_ = 6.3%, tr.BV_k_ = 3.7%). The relative volume of bone marrow in the presence of nanodispersed cerium dioxide is 1.7 times less than in defects without nanodispersed cerium dioxide (with a difference of 11.2%) (MaV_a_ = 16.4%, MaV_k_ = 27.6%). The relative volume of tooth and periodontal tissues is 3.6% lower or 1.1 times less in the presence of nanodispersed cerium dioxide than in a defect without it (Th.V_a_ = 51.6%, Th.V_k_ = 55.2%).

*The structure of the cortical plate regenerated bone tissue 4 weeks after the formation of a bone defect and introduction of suspensions of calcium hydroxide alone and calcium hydroxide with nanodispersed cerium dioxide*.

**Figure 19:**
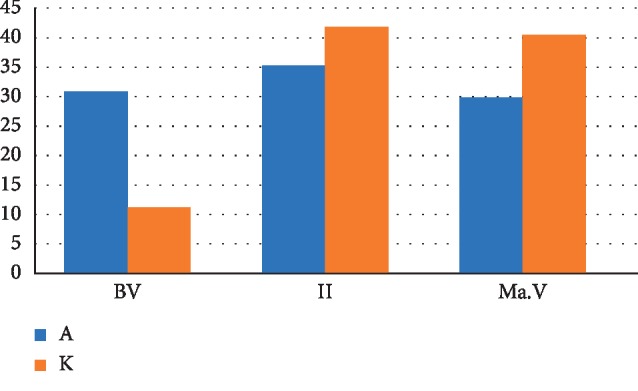
The distribution chart of the structural components of the cortical plate regenerated bone tissue 4 weeks after the formation of a bone defect and introduction of a suspension of calcium hydroxide with and without nanodispersed cerium dioxide. nBv is the relative volume of the newly formed bone tissue(%), II is the relative volume of the granulation tissue (%), and MatV is the relative volume of the material (%) (A—with cerium dioxide and K—without cerium dioxide).

The chart (Figure 19) shows a significant difference in the distribution of the structural components of the cortical plate reganerated bone tissue 4 weeks after the formation of a bone defect and introduction of suspensions of calcium hydroxide alone and calcium hydroxide with nanodispersed cerium dioxide. The relative volume of newly formed bone tissue in the presence of nanodispersed cerium dioxide 2.7 times exceeds that without nanodispersed cerium dioxide, with a difference of 19.5% (nBV_a_ = 30.8%, nBV_k_ = 11.3%). The relative volume of granulation tissue formed in the bone defect in the presence of nanodispersed cerium dioxide is 1.2 times less than in defects without it, with the difference of 6.5% (II_a_ = 35.3%, II_k_ = 41.8%). After 4 weeks, the remainder of the introduced material with nanodispersed cerium dioxide in the bone defect is less by 1.4 times than in the bone defect without nanodispersed cerium dioxide (with a difference of 10.6%) (MaV_a_ = 29.9%, MaV_k_ = 40.5%).

The structure of the regenerated cancellous bone tissue 4 weeks after the formation of a bone defect and introduction of suspensions of calcium hydroxide alone and calcium hydroxide with nanodispersed cerium dioxide.

**Figure 20:**
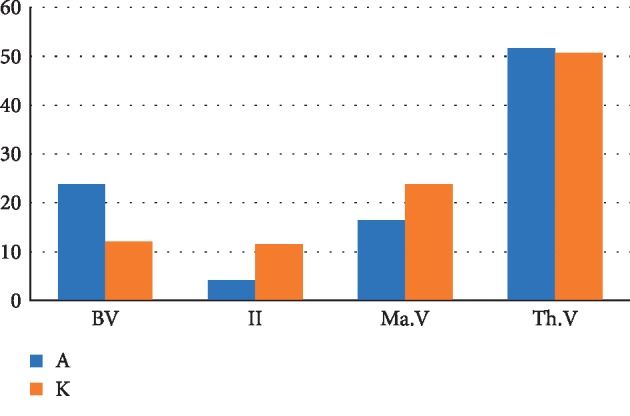
The distribution chart of structural components of regenerated cancellous bone tissue 4 weeks after the formation of a bone defect and introduction of suspensions of calcium hydroxide alone and calcium hydroxide with nanodispersed cerium dioxide. Where Bv is the relative volume of the newly formed bone tissue (%), II is the relative volume of granulation tissue (%), MaV is the relative volume of the bone marrow (%), and Th.V is the relative volume of the tooth and periodontal tissue (%) (A—with cerium dioxide and K—without cerium dioxide).

The chart (Figure 20) reveals a difference in the distribution of the structural components of the regenerated cancellous bone tissue 4 weeks after the formation of a bone defect and introduction of a suspension of calcium hydroxide alone and calcium hydroxide with nanocerium dioxide. The relative volume of the newly formed bone tissue in the presence of nanodispersed cerium dioxide significantly (2 times) exceeds the relative volume of the newly formed bone tissue in the absence of CeO_2_, with the difference of 11.8% (nBV_a_ = 23.9%, nBV_k_ = 12.1%). The relative volume of granulation tissue formed in the bone defect in the presence of nanodispersed cerium dioxide is 2.7 times lower than in defects without it, with the difference of 7.3% (II_a_ = 4.2%, II_k_ = 11.5%). The relative volume of bone marrow in the presence of nanodispersed cerium dioxide is 1.5 times lower than in defects without it (with the difference of 7.4%) (MaV_a_ = 16.4%, MaV_k_ = 23.8%). The relative volume of restored tooth and periodontal tissues is 1% higher in the presence of nanodispersed cerium dioxide than in a defect without it (Th.V_a_ = 51.6%, Th.V_k_ = 50.7%).

The state of interalveolar septum 4 weeks after the formation of a bone defect and introduction of suspensions of calcium hydroxide alone and calcium hydroxide with nanodispersed cerium dioxide.

**Figure 21:**
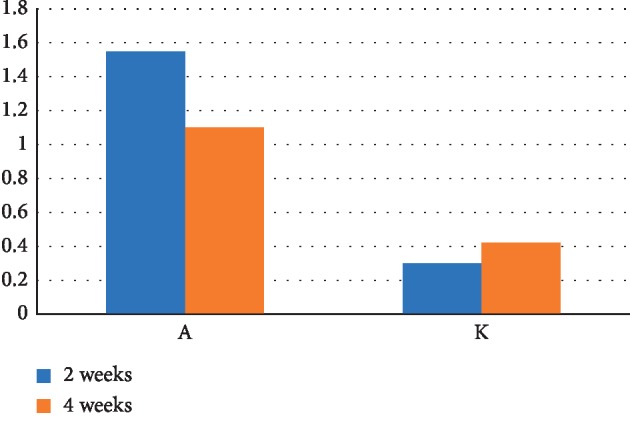
Dynamics of bone formation on the surface of the interalveolar septa after the formation of a bone tissue defect and introduction of suspensions of calcium hydroxide alone and calcium hydroxide with nanodispersed cerium dioxide (A—with cerium dioxide and K—without cerium dioxide).

There are differences in the dynamics of bone formation in the bone tissue defect on the surface of the interalveolar septum resulting from introduction of either a suspensions of calcium hydroxide alone or calcium hydroxide with nanodispersed cerium dioxide (Figure 21). In the early periods of regeneration (2 weeks), the thickness of the newly formed interalveolar partition in the presence of nanodispersed cerium dioxide occurs faster (1.55 mm) than without nanodispersed cerium dioxide (1.1 mm). In later periods (4 weeks), the thickness of the newly formed interalveolar septum in the presence of nanodispersed cerium dioxide (0.3 mm) and 0.42 mm without nanodispersed cerium dioxide. Thus, the regeneration in latter periods (4 weeks) slows down, but in the presence of nanodispersed cerium dioxide, it remains activated.

## 4. Discussion

Experimental in vivo studies were carried out on animals (21 rabbits of the chinchilla breed). Each animal had artificially formed bone defects in the lower jaw. A routine, but minimally invasive surgical protocol, was followed in all cases. Bone defects were created on both sides of the jaw. Calcium hydroxide suspension was chosen as the material replacing the defect, since this material is compatible with nanodispersed cerium dioxide. The formed defects on one side of the jaw were filled with a suspension of calcium hydroxide. A suspension of calcium hydroxide with nanodispersed cerium dioxide was used on the other side of the jaw. The wounds were then sutured. This approach was used to determine the role of nanocrystalline cerium dioxide in the early stages of the course of bone tissue regeneration. Wound healing in all cases occurred by primary intention.

According to histological studies and the results of morphometry, the process and the result of the bone formation in bone defects of the mandible occurred with significant differences on different sides of the jaw (i.e., dependent on presence of nanodispersed cerium dioxide). The results of our studies are consistent with the results of studies in which it was found that *in vitro* CeO_2_ nanoparticles affect the function of proliferation, differentiation, and mineralization of primary osteoblasts. Previous studies have shown that CeO_2_ nanoparticles do not have an acute cytotoxic effect on osteoblasts and may contribute to osteogenic differentiation and mineralization of osteoblasts [3, 6]. In our study, 2 weeks after the formation of the defect, the noticeable differences were revealed in the formation of bone tissue in the defects on different sides of the lower jaw. In the presence of nanocrystalline cerium dioxide, the cortical plate showed intensive increase in the volume of newly formed bone tissue (nBV_a_ = 7.8% and nBV_k_ = 12.3%), less intensive formation of granulation tissue (II_a_ = 39.2% and II_k_ = 42.5%), and more intensive loss of material introduced into the defect (MatV_a_ = 41% and MatV_k_ = 44.3%). After 2 weeks, in presence of nanocrystalline cerium dioxide, the volume of the newly formed bone tissue in the cancellous bone is nBV_a_ = 11.9% and of the granulation tissue is II_a_ = 12.8%, which is significantly higher than without nanocrystalline cerium dioxide: nBV_k_ = 1.8% and II_k_ = 1.8%. In presence of nanocrystalline cerium dioxide, the bone marrow volume is MaV_a_ = 16.4% and tooth and periodontal tissue volume is Th.V_a_ = 51.6%, which is less than without nanocrystalline cerium dioxide: MaV_k_ = 27.6% and Th.V_k_ = 55.2%.

After 4 weeks, differences in bone formation in the defects on different sides of the mandible were also noticeable. The presence of nanocrystalline cerium dioxide continues to influence the course of bone regeneration during this period. The increase in the volume of the newly formed bone tissue and the decrease of granulation tissue and the material introduced into the defect are more intense in the presence of nanocrystalline cerium dioxide: nBV_a_ = 30.8%, II_a_ = 35.3%, and MaV_a_ = 29.9%. Same processes are less intense without nanocrystalline cerium dioxide: nBV_k_ = 11.3%, II_k_ = 41.8%, and MaV_k_ = 40.5%.

The dynamics of bone formation on the surface of the interalveolar septum with introduction of a suspension of calcium hydroxide with nanodispersed cerium dioxide is more vigorous than in absence of nanodispersed cerium dioxide. Other researchers [9] proved (in vitro) that CeO_2_ nanoparticles stimulated the differentiation of osteoblasts. CeO_2_ nanoparticles supported adipogenic transdifferentiation of osteoblasts and the formation of mineralized matrix nodules of osteoblasts. In addition [10], CeO_2_ nanoparticles partially slow down the inflammatory response caused by the corresponding amounts of interleukin 1-alpha.

When using just calcium hydroxide, the reparative osteogenesis is characterized by a moderate course with a tendency to gradual closure of the defect of the cortical plate. By the time of 4 weeks, the defect does not close. In contrast, when using calcium hydroxide with nanodispersed cerium dioxide, the nature of osteogenesis should be characterized as activated. As a result, along with the closure of the bone defect of the outer cortical plate, reparative osteogenesis extends to periodontal tissue and dentin of damaged teeth. In the periodontal tissues, the interalveolar partitions are thickened due to the stratification of the osteoid over the existing lamellar bone tissue, as well as the process of formation of reticular fibrous bone tissue next to the osteoid. In case of damage to the dentin of the roots of the teeth, dentinogenesis occurs with the formation of a combined dentin and bone regenerates. We did not find similar studies by other researchers. The authors plan further in vivo studies to study the biochemical processes observed in the destruction of dentin. It is known that CeO_2_ nanoparticles participate in the redox process and can act as oxidoreductases—enzymes that regulate this process in biological systems [3, 4]. We suggest that this process may affect the plastic function of odontoblasts and stimulate dentinogenesis. The authors plan to study in vivo the regulation of CeO_2_ nanoparticles by reactive oxygen species (ROS), the concentration of enzymes under oxidative stress (catalase and peroxidase), inflammatory cytokines (interleukins (IL)), and nitrogen metabolites (NO), which can affect the process of dentinogenesis.

## 5. Conclusion

Thus, during the formation of a penetrating defect of the outer cortical plate of the mandible bone tissue in a rabbit and introduction of a suspension of calcium hydroxide with nanodispersed cerium dioxide and without it, a significant difference was found during reparative osteogenesis. A morphometric study revealed that the use of nanodispersed cerium dioxide leads to a decrease in the negative effect of inflammation on reparative osteogenesis. The improved osteogenesis is manifested by a significant increase in the newly formed bone substance in the regenerate and, as a result, thickening of the interalveolar partition.

## Data Availability

The histological data used to support the findings of this study may be released upon application to the N. N. Priorov National Medical Research Center of Traumatology and Orthopedics, Moscow, Russia, who can be contacted at alex.volkoff@e-mail.ru and A. I. Evdokimov Moscow State University of Medicine and Dentistry, Department of Therapeutic Dentistry, who can be contacted at lukantik@gmail.com.
